# Annual and Seasonal Variations in Aflatoxin M1 in Milk: Updated Health Risk Assessment in Serbia

**DOI:** 10.3390/toxins17110544

**Published:** 2025-11-02

**Authors:** Saša Krstović, Sandra Jakšić, Jelena Miljanić, Borislav Iličić, Milica Živkov Baloš, Darko Guljaš, Marko Damjanović, Igor Jajić

**Affiliations:** 1 Faculty of Agriculture, University of Novi Sad, 21000 Novi Sad, Serbia; borislav.ilicic@stocarstvo.edu.rs (B.I.); darko.guljas@stocarstvo.edu.rs (D.G.); marko.damjanovic@stocarstvo.edu.rs (M.D.); igor.jajic@stocarstvo.edu.rs (I.J.); 2 Scientific Veterinary Institute “Novi Sad”, 21000 Novi Sad, Serbia; sandra@niv.ns.ac.rs (S.J.); milica@niv.ns.ac.rs (M.Ž.B.); 3 Institute of Food Technology (FINS), University of Novi Sad, 21000 Novi Sad, Serbia; jelena.miljanic@fins.uns.ac.rs

**Keywords:** consumer health, climate impact, risk mitigation, ELISA, food regulations

## Abstract

Aflatoxin M1 (AFM1), a hepatocarcinogenic metabolite of aflatoxin B1, poses significant risks to human health through its presence in milk and dairy products. This study investigates AFM1 contamination in raw milk produced in Serbia from 2021 to 2025, assessing annual and seasonal variations and associated health risks. A total of 907 milk samples were analyzed using enzyme-linked immunosorbent assay (ELISA), revealing contamination in 70.1% of samples, with mean concentrations exceeding the EU regulatory limit of 50 ng/kg. Seasonal analysis identified the highest contamination levels during winter, attributed to increased use of contaminated feed during colder months. Health risk assessments estimated the daily intake of AFM1 and associated health risks, with high-exposure individuals showing notably reduced margins of safety. The research demonstrates the essential requirement for better feed quality management alongside enhanced regulatory oversight along with health programs that reduce AFM1 exposure in Serbian populations.

## 1. Introduction

The importance of mycotoxins as food and feed contaminants has been the focus of both primary producers and processors for the past few decades. One of the most important mycotoxins are aflatoxins (AF) since they can contaminate milk and milk products, among other food and feed [1]. Moreover, the International Agency for Research of Cancer categorized aflatoxin M1 (AFM1) to Group 1 as a proven carcinogen to humans [2]. Contamination of milk and milk products usually occurs by indirect contamination via contaminated feed [3,4], with the carry-over rate from feed (aflatoxin B1-AFB1) to milk (AFM1) being between 0.3 and 6.2% [5]. In cows, AFB1 is converted into AFM1 within 12–24 h after ingestion of contaminated feed, exponentially increasing and reaching maximum after a few days [3,6]. After exclusion of contaminated feed from diets, the AFM1 was undetectable after 72 h [7,8].

Many countries have set or proposed maximum limits (MLs) for AFM1 in milk and AFB1 in animal feed in an effort to reduce exposure risks. The European Union (EU) currently enforces a maximum level of 5 μg/kg for feed for dairy animals, although legal limits for AFB1 in feed vary widely throughout the world [9]. In Serbia, proposed ML for AFB1 has been partially aligned with the EU since April 2014 [10]. Regarding the AFM1 regulation, the EU has established ML in raw milk of 50 ng/kg [11]. In the United States [12], the ML is set at 500 ng/kg, which is also the case in several Asian countries, including China, and in South American countries, such as Brazil. In Serbia, the regulation on AFM1 levels in milk was first aligned with EU standards in 2011, following the adoption of the new regulation on maximum permissible residues in food and feed [13]. Since then, the AFM1 ML has been revised several times, and the current version of the regulation sets the ML at 250 ng/kg in milk.

Italy was one of the first EU countries to face the problem of milk contamination with AFM1 [14]. The problem has been addressed by far-reaching measures to avoid the continuation of the problem [15,16]. However, significant flaws in the control system were exposed in Serbia during the 2013 aflatoxin crisis. Widespread AFM1 contamination of raw milk resulted from abnormally high AFB1 levels in maize caused by prolonged drought and poor storage conditions [17]. To avoid throwing away large amounts of milk, authorities temporarily raised the national maximum residue limit for AFM1 from the EU standard of 50 ng/kg to 500 ng/kg. This decision was strongly criticized by consumer advocacy groups and public health experts [18]. Consumer confidence was damaged by this regulatory relaxation and the fragmented feed and milk monitoring, which also made it clear that a stronger risk-management system was required [18].

In this study, we use raw milk as an indicator of AFM1 exposure due to its role as a primary medium for AFM1 contamination. Heat treatments like pasteurization and UHT processing do not significantly affect AFM1 concentrations due to its inherent heat stability [19]. Published studies have shown variable results regarding AFM1 reduction during different stages of milk product processing, which may be attributed to differences in initial contamination levels, temperature ranges, and analytical methods used for AF determination [20,21]. Raw milk is a good indicator for determining AFM1 exposure in dairy products because it is the source of contamination prior to processing and its contamination levels are less impacted by heat treatments. Because of this, raw milk serves as a valuable starting point for assessing AFM1 contamination throughout the larger dairy supply chain.

This study aims to assess the occurrence, annual and seasonal variations, and associated health risks of AFM1 contamination in Serbian raw milk from 2021 to 2025. This study analyzes the factors influencing AFM1 levels, including climatic conditions and feeding practices, to provide an assessment of contamination dynamics and propose recommendations for mitigating public health risks.

## 2. Results and Discussion

This study aimed to examine the concentration of AFM1 in cow’s milk across different years and seasons to assess variations and potential risks. A total of 907 milk samples were analyzed from 2021 to 2025. The results were expressed as least square means (LSM), standard error (SE), including minimum and maximum values with 95% confidence intervals.

### 2.1. Annual Variations

**Figure 1:**
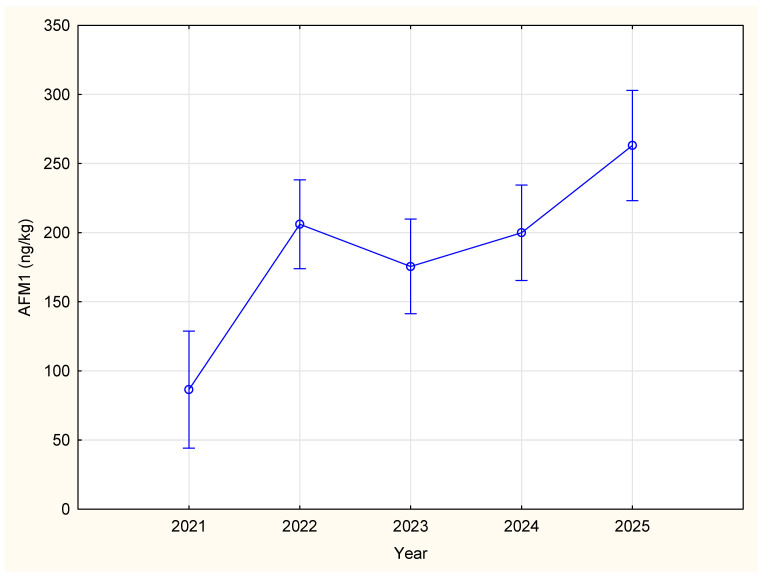
Annual variations of AFM1 content in the 2021–2025 period in Serbia (F (4, 902) = 9.49; *p* < 0.05; Vertical bars denote 0,95 confidence intervals).

Table 1 and Figure 1 summarize the annual variations in AFM1 concentrations during the studied period. The number of milk samples analyzed for AFM1 concentrations varied across the years 132 in 2021, 228 in 2022, 201 in 2023, 198 in 2024, and 148 in 2025. The highest mean concentration of AFM1 (263.05 ng/kg) was detected in 2025, with levels ranging from 223.12 to 302.99 ng/kg. On contrary, the lowest concentration of AFM1 was recorded in samples from 2021, with a mean value of 86.40 ng/kg (range: 44.11–128.69 ng/kg).

Based on the results of the ANOVA, it was determined that there was a significant difference for AFM1 in cow’s milk throughout different years (F (4, 902) = 9.49; *p* < 0.05). Further statistical analysis (Duncan’s post hoc test) revealed that the mean concentration of AFM1 observed in 2025 (263.05 ng/kg) was significantly higher than in the other monitored years (*p* < 0.05). In contrast, samples from 2021 showed a significantly lower mean AFM1 concentration (86.40 ng/kg) when compared to samples from other observed years (*p* < 0.05). On the other hand, samples from the period 2022 to 2024 showed intermediate levels when compared to 2021 and 2025, but there was no significant annual variation among them (*p* > 0.05).

The observed variation in AFM1 contamination levels in milk can be attributed to differences in maize contamination with AFs. AFM1 is a metabolic derivative of AFB1, which contaminates maize crops during their pollination and grain-filling phases [22], typically occurring from July to August.

Meteorological data provided by the Republic Hydrometeorological Service of Serbia (RHSS) [23,24,25,26,27,28], indicate that weather conditions during the critical July–August period varied significantly between the years. In 2020, which experienced no major temperature extremes that could disrupt phenological processes in plants, conditions were favorable for maize growth. On the other hand, the weather conditions in 2021 and 2023 were less favorable for crops, but there was a significant amount of precipitation. In contrast, 2022 and 2024 were characterized by a warm and dry climate during July and August, although some precipitation occurred during the critical dry period (e.g., beginning and end of July), but not in August. These weather patterns likely played a key role in maize contamination with AFB1, contributing to elevated AFM1 levels in milk in 2022, 2024, and 2025, compared to 2021 and 2023. Additionally, analyzing the weather conditions for the year 2025, which included three heat waves and three cooling periods in July, combined with a period of highly unfavorable rainfall, the condition of crops and the soil itself continued to worsen throughout most of August, leading to substantial damage to crops, particularly maize and soy. This may be a strong indication that AFM1 levels are likely to be high in the coming period, stressing the importance of implementing preventive measures.

Considering that Serbia has a moderate continental type of climate, there has been a general understanding that only a low risk of AFs contamination is present [18]. Until 2012, there were numerous publications about the presence of AFs in different feed and food products with low [29,30,31,32]. Due to climate change and the development of advanced methods of its detection, the AF occurrence became more frequent. During the last years, the presence of AFM1 in milk and dairy products has been the subject of numerous studies: more than 270 research articles and more than 170 review papers have been indexed in the Web of Science database during the last five years [1]. From 2012 until now, contamination with AFs in Serbia from different foods and feeds has been repeated regularly. Contamination of milk and dairy products with AFM1 occurred at the very beginning of 2013, as a consequence of maize contamination with aflatoxins, which occurred in 2012 and was characterized by drought conditions [33]. In the period from 2013 to 2016, the total frequency of the AFM1 contaminant was 67.8% based on 10,781 analyzed samples, where 27.6% of the samples were above the prescribed values [34]. The same author conducted a study from 2015 to 2023, including about 14,000 milk samples, and found that 78% of milk and 42–79% of different dairy products were contaminated with AFM1 [35]. Among the most comprehensive investigations in Serbia was the one carried out by Milićević et al. [36], who analyzed the period from 2015 to 2018 with 20,232 milk samples and found a high prevalence of AFM1 with 70%, 85%, 78.7%, and 82.4%, respectively.

As previously mentioned, higher levels of AFM1 in the years 2025, 2024, and 2022 can be attributed to adverse weather conditions. Pleadin et al. [37] stated that the occurrence of AFs in maize is mainly attributed to the droughts, suggesting Palmer Drought Z-index (moisture anomaly indicator) as one of the relevant indicators of drought. After analyzing the drought indicator (Z-index) provided by the RHSS [23,24,25,26,27,28], it is evident that a significant portion of Serbia experienced moderate to extreme drought in July and August 2022, 2024, and 2025, which corresponds with our results.

### 2.2. Seasonal Variations

**Figure 2:**
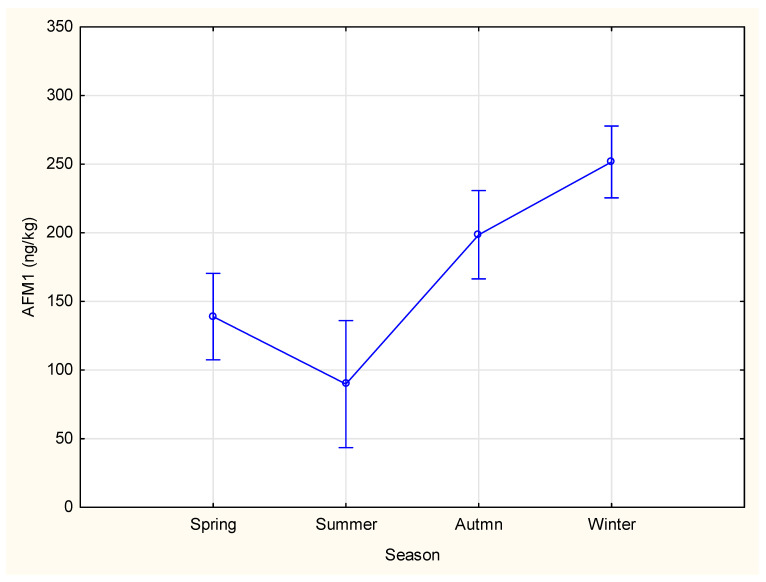
Seasonal variations of AFM1 content in the 2021–2025 period in Serbia (F (3, 903) = 16.61; *p* < 0.05; Vertical bars denote 0,95 confidence intervals).

Regarding the AFM1 variation during different seasons of the year, the number of milk samples analyzed was 235 for spring, 109 for summer, 224 for winter, and 339 for autumn. Seasonal analysis, as indicated in Table 2 and visualized in Figure 2, revealed that the highest concentration of AFM1 in milk samples occurred during winter, with a mean value of 251.60 ng/kg (range: 225.39–277.81 ng/kg). In contrast, the lowest concentration of AFM1 was recorded in the summer, with a mean level of 89.74 ng/kg (range: 43.52–135.97 ng/kg).

ANOVA analysis revealed that the season had a significant impact on AFM1 levels in cow’s milk (F (3, 903) = 16.61; *p* < 0.05). Duncan’s post hoc analysis indicated that AFM1 concentrations in milk were significantly different among seasons (*p* < 0.05), except spring and summer, which were not significantly different from one another (*p* > 0.05). Moreover, spring and summer showed significantly lower levels when compared to autumn and winter (*p* < 0.05). Surprisingly, samples from winter season were significantly higher than other seasons (*p* < 0.05).

This seasonal variation in AFM1 levels is likely due to variations in the types of feed consumed by cows during colder months. During winter, the reduced availability of fresh green forage often leads to an increased reliance on mixed complementary feed, such as legumes and maize. This shift in diet can elevate exposure to AFB1 in contaminated feed, subsequently increasing AFM1 concentrations in milk. Bilandžić et al. [38] noted that higher AFM1 levels during winter are associated with increased use of stored and processed feed, in contrast to the summer months, when cows consume more fresh raw feed. Similar observations were reported by Tomašević et al. [39], who indicated significantly higher levels of AFM1 in raw milk samples from winter (358 ng/kg) and spring (375 ng/kg) when compared to autumn (103 ng/kg) and summer (39 ng/kg), which was in line with our results for winter but not for spring and autumn seasons. Studies on AFM1 occurrence also support our findings. Miočinović et al. [40] established the highest concentration of AFM1 in autumn, with 44.93% of all raw milk samples exceeding the EU ML. Examining 248 raw milk samples from 2021, Jauković et al. [41] found that AFM1 concentrations were below the detection limit in March, April, June, and July. In contrast, contamination was detected in 27.27% of samples in September, 27.59% in October, 75.0% in November, and 62.5% in December. Đekić et al. [42] determined that the highest number of raw milk samples with AFM1 was obtained during autumn, whereas the lowest number was obtained during summer.

Temperatures in the Republic of Serbia are expected to rise continuously during this century, with averages projected to be 3 to 5 °C higher than mid-20th century averages [43]. Given that the three hottest years in Serbia since 1951 have so far been 2024, 2012, and 2025 [27], respectively, it is imperative to create a thorough surveillance, control, and response plan to track aflatoxins across the whole food chain and avoid a repeat of the 2012 incident, in which abnormally high aflatoxin levels were found in milk. One example of such a system is found in Italy, where the recent decrease in AF positivity has been attributed to the implementation of the Regional Surveillance Plan. This plan addressed the contamination crisis and introduced measures to reduce and maintain average AFM1 levels at a low threshold [44].

### 2.3. Health Risk Assessment

Health risk assessment was based on the average daily consumption of milk is 0.135 kg per person [45]. Body weight in adults (15+ years): 76.27 kg [46]. Daily consumption MoE calculations were based on benchmark dose (BMDL_10_) of 400 ng/kg body weight per day for AFB1 and a potency factor of 0.1 for AFM1 [47].

Table 3 shows that out of the 907 samples analyzed, 636 samples (70.1%) tested above the EU legal limit of 50 ng/kg for AFM1. The LB mean concentration of AFM1 was 189.8 ng/kg, while the UB mean was 190.4 ng/kg. The median or P50 concentration was at 98 ng/kg, whereas P90 and P95 concentrations stood at 491 ng/kg and 684 ng/kg. The EDI of AFM1 varied from a median value of 0.336 ng/kg b.w. to 1.211 ng/kg b.w. at the 95th percentile. The derived MoE values from these intakes ranged from a maximum of 23,060 (P50) to a minimum of 3304 (P95), depicting decreasing safety margins with increasing AFM1 exposure.

The results indicate a large proportion (70.1%) of AFM1-contaminated raw milk samples taken in Serbia, reflecting ongoing challenges in sustaining compliance with aflatoxin safety standards for milk and dairy products. AFM1 levels (LB: 189.8 ng/kg, UB: 190.4 ng/kg) are above the EU’s regulatory standard of 50 ng/kg for AFM1 in raw milk, suggesting potential health risks to consumers.

The primary contributors to total average AFM1 exposure across all age groups are “liquid milk” and “fermented milk products” [47]. The presence of AFM1 in dairy products poses a public hazard due to its well-documented adverse effects on human health [2]. Although AFs are recognized as genotoxic carcinogens, neither the FAO/WHO Joint Expert Committee on Food Additives (JECFA) nor the European Community’s Scientific Committee on Food (SCF) has established a tolerable daily intake (TDI) for AFM1 in human diet. Kuiper-Goodman [48] established maximum TDI of 0.2 ng/kg b.w. per day for AFM1 in male rats.

While the EDI for AFM1 through milk consumption is already a concern at the median level (0.173 ng/kg b.w.), it is significantly higher and increases rapidly at higher percentiles (P95 = 1.211 ng/kg b.w.). This shows that even average consumption may pose significant health risk; high consumers of dairy products would be even more at risk. The chronic dietary exposure to AFM1 in the total adult population across European countries, according to EFSA [47], is between 0.05 and 0.06 ng/kg b.w. per day for mean exposure and 0.13 and 0.16 ng/kg b.w. per day for p95 percentile exposure.

Udovički et al. [35] observed the highest exposure in children with mean AFM1 EDI of 0.336 ng/kg b.w. per day, followed by adolescents with 0.183 ng/kg b.w. per day, then adult females with mean EDI of 0.161 ng/kg b.w. per day and adult males with 0.126 ng/kg b.w. per day. The same authors reported that the margin of exposure, based on mean EDI across all population groups, exceeded the risk-associated threshold, indicating generally low health risks from AFM1 exposure for the overall population. However, they stressed that the risk is not negligible, particularly for children, who represent a larger proportion of individuals exposed to AFM1 levels associated with potential health effects.

The MoE analysis provides a measure of potential risk, which links a toxicological reference point to estimated human exposure. Following the EFSA guidance [47], values of MoE below 10,000 are a concern for health impacts of potential genotoxic and carcinogenic substances, including aflatoxins. The mean (LB = 11,904; UB = 11,871) and median (P50 = 23,060) MoE values in the study indicate a low risk for the adult population. However, the MoE levels were concerning at 75th percentile (9912) and quite lower at the 90th percentile (4603), and 95th percentile (3304) indicating there is a high risk for consumers incurring elevated exposures to aflatoxins. The low MoE values at P90 also correlate to an elevated risk for the low-dose carcinogenic agent for hepatocellular carcinoma. This is particularly important for infants whose primary diet consists of milk and dairy products, and for whom it has been established that they have the highest estimated chronic dietary exposure to AFM1 [47].

The high level of contamination and high AFM1 levels for upper percentiles suggests there might be a lack of feed quality or storage, since AFM1 in the milk is derived from AFB1 contaminated feed [4], as AFB1 concentration in contaminated feed result in high AFM1 levels in milk and dairy foods [49]. Inadequate feed storage and absence of permanent feed monitoring could elevate AFM1 levels in raw milk [40]. A similar conclusion was observed by Xiong et al. [50] who indicated that during winter, the lack of fresh green feed forces dairy farmers to feed cows substantial amounts of stored or conserved feeds (such as corn, cottonseed and silage), which potentially could contain AFB1 under inadequate storage conditions. The study also indicates that contamination is seasonally variable, which trends towards more exposure during certain timeframes, like winter when diet shifts in dairy cows. Moreover, our findings align with previous studies that have reported increased AFM1 contamination during colder seasons [39,41,49,50,51]. Contrary to these findings, Özbey [52] established that the highest concentration was during the summer. Furthermore, the author stated that the elevated AFM1 concentrations observed in the summer were due to the limited pasture areas availability, shepherd challenges in rural areas, the composition of diet (silage, mixed feed, pulp, roughage), restricted grazing time, and storage conditions such as humidity and temperature. Moreover, along with AFM1, there are reports of multiple fungal toxins such as ochratoxin A, zearalenone, or emerging mycotoxins (for example, beauvericin, enniatins) in Serbian maize that may affect dairy production chains [53]. In addition, combined exposures could result in either additive or synergistic toxic effects, based on recent EFSA opinions and in vivo studies [47] Therefore, future monitoring programs should adopt multi-mycotoxin screening approaches to assess overall dietary exposure risks and consumer protection more accurately.

### 2.4. Limitations

There are several limitations in this study that ought to be considered. The first limitation is that the concentrations of AFM1 were determined by ELISA, which is a widely acknowledged quantitative screening technique, but there was no confirmatory analysis by LC-MS/MS. Moreover, feed or total mixed ration (TMR) samples were not collected regularly which reduced the possibility of milk contamination being directly traced back to feed contamination. The sampling was meant to include all areas and seasons in Serbia, yet a fully structured farm-level sampling protocol that encompassed each farm, season, and feed material could not be set up due to the study being of a large scale and limited resources. Lastly, the assessment of exposure for the population did not include infants and children due to insufficient data on milk intake for these groups. It is hoped that future research will be able to eliminate these limitations by using feed analysis, more detailed farm-level sampling, and including vulnerable population groups in risk assessment.

## 3. Conclusions

Our study as the multi-year survey (2021–2025) on AFM1 contamination in Serbian raw milk confirms that contamination still exists as a serious problem. Out of 907 analyzed samples 70.1% were higher than the EU maximum limit and mean concentrations ranged from 86.4 ng/kg in 2021 to 263.0 ng/kg in 2025. There was a large seasonal impact with AFM1 levels highest in the winter (251.6 ng/kg) and lowest in the summer (89.7 ng/kg) due to the increased use of stored feed during the winter months.

The health risk assessment corresponded to a median estimated daily intake (EDI) of 0.173 ng/kg b.w. day^−1^ and a margin of exposure (MoE) of 23,060 suggesting overall low concern for the general adult population. Relevance of risk exists at the 95th percentile (1.211 ng/kg b.w. day^−1^; MoE = 3304) in relation to higher consumption dairy product eaters and vulnerable populations such as children.

Climatic variability characterized by frequent droughts and high summer temperatures was significantly associated with an increase in AFM1 presence highlighting the progressive nature of climate change on feed contamination and the need for risk management adaptation and measures. Strengthening national surveillance systems and working towards integrated European multi-toxin monitoring for consumer protection and compliance with EU food safety standards in future climatic scenarios will be necessary.

## 4. Materials and Methods

### 4.1. Sample Collection

A total of 907 raw milk samples were collected in the period from January 2021 to August 2025 and analyzed for the content of AFM1. Milk samples were collected directly from individual farmers across Serbia. The raw milk samples were collected under the umbrella of the official dairy herd improvement and milk quality control program. Milk collection in this system is organized by a regular schedule of lines for pickup and transport that cover all sub-regions. Milk samples are collected regularly from dairy farms, year-round. The raw milk samples selected for this study were randomly selected across those routine samplings for analysis of AFM1. Sampling was completed in a way to ensure geographic area (as dairy farms were being sampled from individual lines representing a collection area) and seasonal representation of samples collected every year. As such, the sampling design allowed for representative coverage of the regional dairy production system rather than specific dairy farms, as there were changes in ownership for a subset of the farms, and in some cases, the farm was no longer in operation during the monitoring period. Sampling procedure was carried out in accordance with EU regulation [54]. On each farm, 3–5 incremental samples were taken from the bulk tank and combined to form a single aggregate sample per sampling. Samples were immediately transported to the laboratory, kept in the refrigerator at 4 °C and analyzed within 24 h.

### 4.2. Sample Analysis

AFM1 in milk was quantified using the Veratox® Aflatoxin M1 ELISA kit (Neogen, Lansing, MI, USA), with absorbance measured at 630 nm (Multiskan FC, Thermo Scientific, Suzhou, China) and analyzed using Rida^®^Soft Win (R-Biopharm, Darmstadt, Germany). The method LOD was 5 ng/kg (25 ng/kg for diluted samples), well below the regulatory limit of 250 ng/kg. Method performance was verified using spiked samples and proficiency tests (FAPAS 04396 and 04490; z-scores 1.4 and −0.6). The method trueness, calculated based on the analysis of six replicates of Fapas T04300QC sample, was 110%, which corresponds to the range recommended by EU regulation [54], while minimal matrix effects cannot be excluded. Method precision was calculated from results generated under repeatability conditions (RSD_r_, H = 36%) and under reproducibility conditions (RSD_R_, H = 54%). Linearity of the method indicated high correlation coefficient across the method range (R^2^ = 0.985).

### 4.3. Health Risk Assessment

The health risk assessment was carried out by using data on AFM1 occurrence and food consumption. To address the uncertainty from undetectable toxin levels (below the limit of detection or LOD), both the lower bound (LB) and upper bound (UB) estimation methods were employed. These approaches are recommended for chemicals likely to be present in food, such as naturally occurring contaminants like mycotoxins. According to the [55,56] substitution methods, values reported below the LOD were assigned either a value of zero (LB) or the LOD value itself (UB). Additionally, selected percentiles (P50 to P95) of AFM1 concentrations were calculated.

Consumption data were taken from the National Statistical Office [45]. Absence of reliable data regarding milk consumption by infants and children in Serbia prevented the exposure assessment for these age groups. Limitations like this should be tackled in the future since these categories are exceptionally vulnerable to AFM1 exposure. The estimated daily intake (EDI) of AFM1 from milk was calculated by combining the selected concentration levels (LB and UB mean, P50, P75, P90, P95) with the respective milk consumption values [57]:

EDI = Σ (Ci × V_w_)/b.w.

where:

EDI = estimated daily intake of AFM1 through milk (ng/kg body weight per day)

Ci = AFM1 concentration level in milk (mean or selected percentile, ng/kg)

V_w_ = consumed amount of milk (kg/day)

b.w. = body weight (kg)

The mean body weight for specific population groups in Serbia was sourced from the national health status survey [46].

Risk characterization was done by estimating the margin of exposure (MoE) [47]. The MoE was calculated as the ratio of the benchmark dose lower confidence limit (BMDL_10_) to the AFM1 exposure from milk consumption:

MoE = BMDL_10_/EDI

where:

MoE = margin of exposure (unitless)

BMDL_10_ = benchmark dose lower confidence interval (400 ng/kg b.w. per day for AFB1; potency factor for AFM1 is 0.1 relative to AFB1)

EDI = estimated daily intake of AFM1 from milk (ng/kg b.w. per day)

A MoE of 10,000 or higher was considered to pose low health risk.

### 4.4. Statistical Methods

Data analysis was carried out in Statistica software version 14.0.0.15 (2020, TIBCO Software Inc., Palo Alto, CA, USA) using one-way ANOVA with Duncan’s post hoc test. Differences were considered significant at *p* < 0.05.

## Figures and Tables

**Table 1:** Annual variations of AFM1 (ng/kg) in the 2021–2025 period (95% confidence interval).

Year	LSM	SE_LSM_	MIN	MAX	*n*
2021	86.40 ^a^	21.55	44.11	128.69	132
2022	206.051 ^b^	16.39	173.87	238.23	228
2023	175.54 ^b^	17.46	141.27	209.81	201
2024	199.95 ^b^	17.59	165.42	234.48	198
2025	263.05 ^c^	20.35	223.12	302.99	148

LSM—least square means, SE_LSM_—standard error, MIN—minimum (−95%), MAX—maximum (+95%), n—number of samples. Values with the same letters are not significantly different (*p* < 0.05).

**Table 2:** Seasonal variations of AFM1 (ng/kg) in the 2021–2025 period (95% confidence interval).

Season	LSM	SE_LSM_	MIN	MAX	*n*
Spring	138.88 ^a^	16.04	107.40	170.36	235
Summer	89.74 ^a^	23.55	43.52	135.97	109
Autumn	198.56 ^b^	16.43	166.32	230.81	224
Winter	251.60 ^c^	13.35	225.39	277.81	339

LSM—least-square means, SE_LSM_—standard error, MIN—minimum (−95%), MAX—maximum (+95%), *n*—number of samples. Values with the same letters are not significantly different (*p* < 0.05).

**Table 3:** AFM1 levels in raw milk, estimated daily intake of AFM1 from milk consumption, and the corresponding margin of exposure for the risk of hepatocellular carcinoma in adults in Serbia.

AFM1 Level	Raw Milk (ng/kg)	EDI (ng/kg b.w.)	MoE
*n*	907		
*n* (%) above ML	636 (70.1)		
LB mean	189.8	0.336	11,904
UB mean	190.4	0.337	11,871
P50	98	0.173	23,060
P75	228	0.404	9912
P90	491	0.869	4603
P95	684	1.211	3304

*n*—number of samples; AFM1—aflatoxin M1; EDI—estimated daily intake; MoE—margin of exposure; ML—maximum level (50 ng/kg); LB—lower bound; UB—upper bound.

## Data Availability

The original contributions presented in this study are included in the article. Further inquiries can be directed to the corresponding author.

## Abbreviations

The following abbreviations are used in this manuscript:

AF	Aflatoxin
AFB1	Aflatoxin B1
AFM1	Aflatoxin M1
b.w.	Body weight (kg)
BMDL_10_	Benchmark dose lower confidence interval (400 ng/kg b.w. per day for AFB1; potency factor for AFM1 is 0.1 relative to AFB1)
Ci	AFM1 concentration level in milk (mean or selected percentile, ng/kg)
DL	Detection Limit
EDI	Estimated Daily Intake
EFSA	European Food Safety Authority
EU	European Union
FAO	Food and Agriculture Organization of the United Nations
IARC	International Agency for Research on Cancer
JECFA	Joint FAO/WHO Expert Committee on Food Additives
LB	Lower Bound
LOD	Limit of Detection
LSM	Least Squares Mean
MAX	Maximum
MIN	Minimum
ML	Maximum Level
MoE	Margin of Exposure
RHSS	Republic Hydrometeorological Service of Serbia
SE	Standard Error
SE_lm_	Standard Error of the Least Squares Mean
TDI	Tolerable Daily Intake
UB	Upper Bound
UHT	Ultra-High Temperature
Vw	Consumed amount of milk (kg/day)
WHO	World Health Organization
